# Epicardial left atrial appendage closure with the lariat device in a patient with atrial septal closure

**DOI:** 10.1002/joa3.12600

**Published:** 2021-08-11

**Authors:** Makoto Sano, Spyridon Liosis, Jan‐Christian Reil, Thomas Fink, Julia Vogler, Charlotte Eitel, Christian‐Hendrik Heeger, Karl‐Heinz Kuck, Roland Richard Tilz

**Affiliations:** ^1^ University Heart Center Lübeck Division of Electrophysiology Medical Clinic Ⅱ University Hospital Schleswig‐Holstein Lübeck Germany; ^2^ Division of Cardiology Internal Medicine Ⅲ Hamamatsu University School of Medicine Hamamatsu Japan

**Keywords:** atrial septal closure, Lariat, left atrial appendage closure

## Abstract

We report a case of percutaneous epicardial left atrial appendage exclusion in a patient with the atrial septal closure

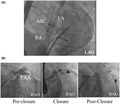

## 1 CASE MANUSCRIPT

Left atrial appendage (LAA) closure has become an alternate therapy to long‐term oral anticoagulation therapy for stroke prevention in patients with atrial fibrillation (AF) and a high bleeding risk. Trans‐septal access following an atrial septum closure (ASC) is challenging and is generally positioned in contra‐indication of endocardial LAA closure. Successful implantation of endocardial LAA occlusion after the ASC has reported on a few cases. We report the first experience of percutaneous epicardial LAA exclusion in a patient with the ASC.

**FIGURE 1:**
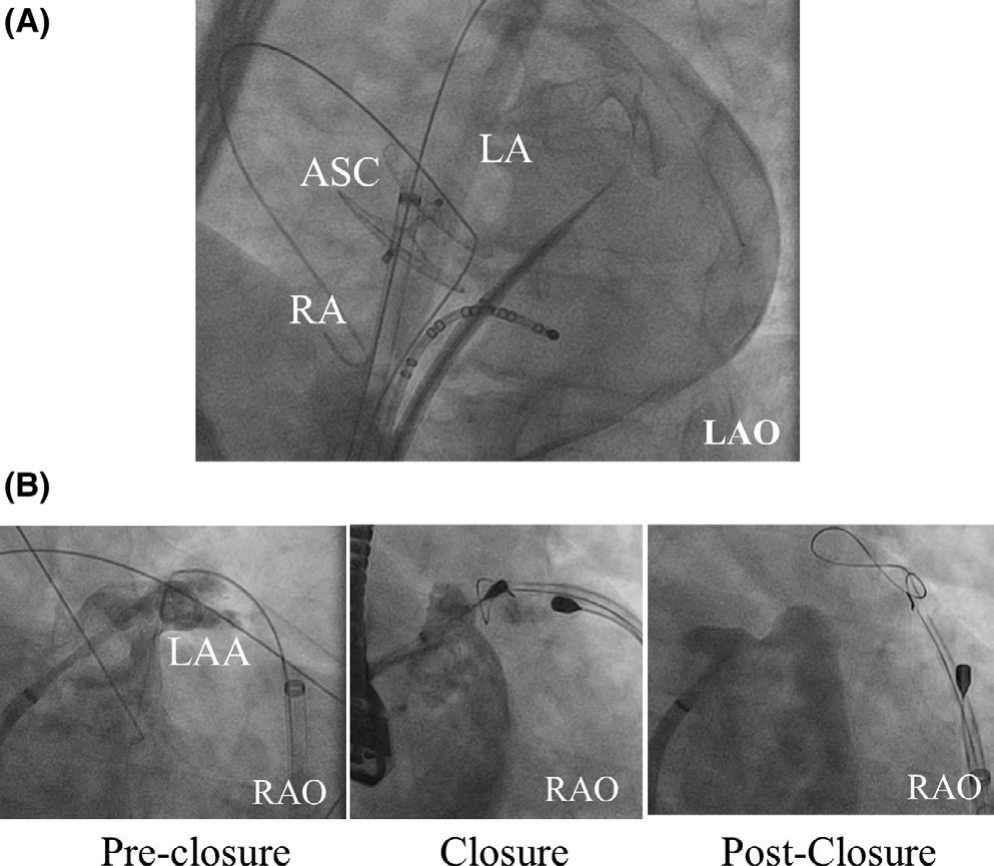
Fluoroscopic images during the epicardial LAA ligation. (A) A trans‐septal puncture was carefully performed under fluoroscopic guidance posteroinferior to the device. (B) LAA was visualized via angiography. The endocardial and epicardial magnetic tip catheters were introduced inside the LAA and to the epicardial LAA aiming for connection at the distal tip of an anterior LAA lobe. The LAA was ligated at the ostial position. LA angiography showed successful LAA ligation. ASC, atrial septal closure device; RA, right atrium; LA, left atrium; LAA, left atrial appendage; LAO, left anterior oblique; RAO, right anterior oblique

A 51‐year‐old man with paroxysmal AF (CHA2DS2‐VASC score; 2) underwent percutaneous ASC (30 mm Amplatzer, Abbott, Illinois, USA) for a patent foramen ovale following a cryptogenic stroke two years before. In addition, an implantable loop recorder (ILR) (REVEAL XT, Medtronic, MN, USA) was implanted. During the follow‐up, frequent AF episodes were documented and transient ischemic attack was observed under oral anti‐coagulation (OAC) therapy. Although lifelong OAC therapy was recommended with mild bleeding risk (HAS‐BLED score of 2, ORBIT‐AF bleeding score of 0), the patient refused long‐term OAC therapy and requested LAA occlusion for stroke prevention instead of a second foreign body. After a great deal of consideration for a procedural limitation of trans‐septal puncture following ASC and a potential risk of the recurrent intra‐atrial shunt, we chose epicardial LAA exclusion using the LARIAT device (SentreHeart, CA, USA), potentially favorable to electrical and structural LAA remodeling decreasing the AF recurrence rate and a smaller sheath reducing the risk for post‐procedural intra‐atrial shunt. Following dry epicardial puncture, a trans‐septal puncture was carefully performed under fluoroscopic guidance posteroinferior to the device using an 8,5F SL 1 sheath (Abbott, IL, USA) (Figure 1A). Chicken wing‐shaped LAA was visualized via angiography in the left anterior oblique (LAO) 40° and right anterior oblique (RAO) 30°/caudal 20° projection (Figure 1B). Fluoroscopic and TEE‐guided LAA closure was successfully performed using the epicardial Lariat device without complication (Figure 1B). The TEE 6 weeks after the procedure showed complete closure of the LAA without device thrombus and without a recurrent intra‐atrial shunt. OAC was replaced with single aspirin 4 months after the procedure along a statement. No arrhythmia recurrence was observed with beta‐blocker, with AF burden decreasing from <5% of pre‐LAA ligation to 0% of post.

LAA closure is an alternative therapy for patients with AF and a high bleeding risk for long‐term anticoagulation therapy. Epicardial LAA occlusion has several potential advantages as compared to endocardial devices. First, the LAA is one of the important substrates and triggers for AF. LAA ligation results in favorable electrical and structural remodeling of the LAA and has been shown to decrease the AF recurrence rate associated with the LAA trigger. Second, a smaller trans‐septal sheath (8F) is required for the LA access thereby reducing the risk for intra‐atrial shunt. Third, no foreign body remains in the heart. LAA ligation is a therapeutic option of LAA closure in a case with the difficulty of trans septal puncture and rhythm management for AF.

## CONFLICT OF INTEREST

R. R. Tilz received travel grants from SentreHeart. The other authors have nothing to disclose.

